# Supplementary material for: Relation of Metal-Binding Property and Selective Toxicity of 8-Hydroxyquinoline Derived Mannich Bases Targeting Multidrug Resistant Cancer Cells
Source: Cancers (Basel). 2021 Jan 5;13(1):154. doi: 10.3390/cancers13010154 (PMC7796460; doi:10.3390/cancers13010154)
Supplement: Supplementary file 1 [file cancers-13-00154-s001.pdf]

RELATION OF METAL-BINDING PROPERTY AND SELECTIVE TOXICITY OF 8-HYDROXYQUINOLINE DERIVED MANNICH BASES TARGETING MULTIDRUG RESISTANT CANCER CELLS

Veronika F.S. Pape[a,†], Anikó Gaál[b], István Szatmári[c], Nóra Kucsma[a], Norbert Szoboszlai[b], Christina Streli[d], Ferenc Fülöp[c], Éva A. Enyedy[e,f*], Gergely Szakács[a,g*]

[a] Institute of Enzymology, Research Centre for Natural Sciences, Hungarian Academy of Sciences, Magyar Tudósok körútja 2, H-1117 Budapest, Hungary
[b] Institute of Chemistry, Eötvös Loránd University, Pázmány Péter sétány 1/A, H-1117 Budapest, Hungary
[c] Institute of Pharmaceutical Chemistry and Stereochemistry Research Group of Hungarian Academy of Sciences, University of Szeged, Eötvös u. 6, H-6720 Szeged, Hungary
[d] Institute of Atomic and Subatomic Physics, Technical University Vienna, Stadionallee 2, A-1020 Vienna, Austria
[e] Department of Inorganic and Analytical Chemistry, Interdisciplinary Excellence Centre, University of Szeged, Dóm tér 7, H-6720 Szeged, Hungary
[f] MTA-SZTE Lendület Functional Metal Complexes Research Group, University of Szeged, Dóm tér 7, H-6720 Szeged, Hungary
[g] Institute of Cancer Research, Medical University of Vienna, Borschkegasse 8a, A-1090 Vienna, Austria

[†] Present address: Department of Physiology, Semmelweis University, Faculty of Medicine, Tűzoltó utca 37-47, H-1094 Budapest, Hungary

SUPPORTING INFORMATION

8-Hydroxyquinoline derived Mannich bases used in this study

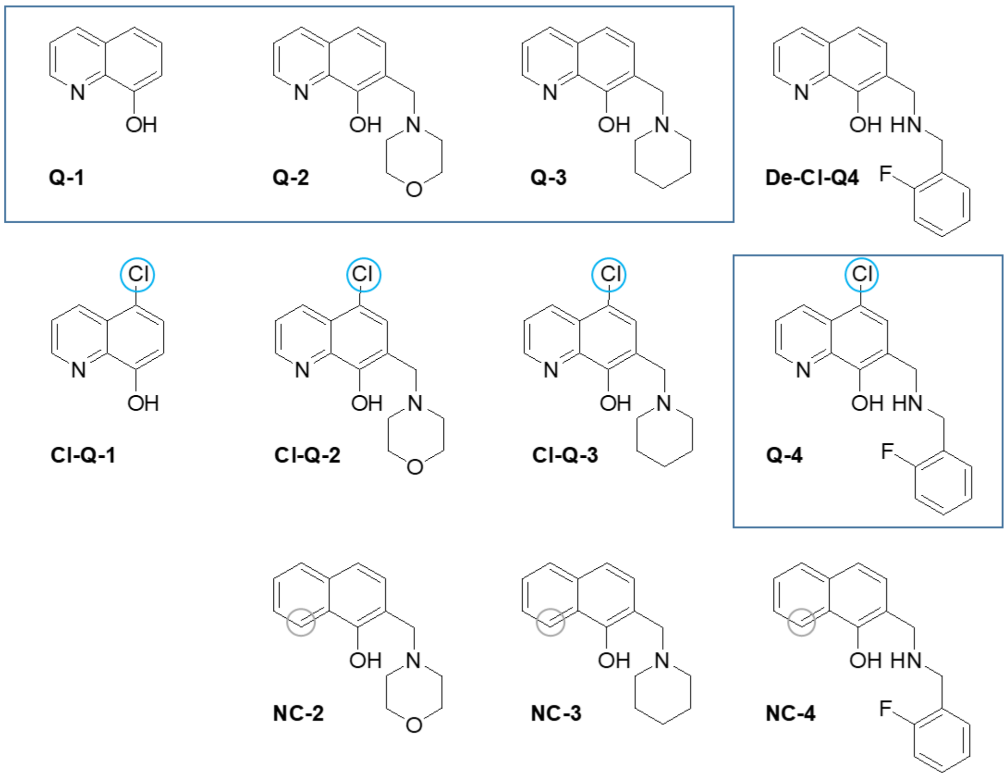

Figure S1: Compounds applied in the study. Compounds that were investigated previously with regard to their iron(III) and copper(II) binding abilities, are framed with boxes. Chlorination in R5 is indicated by blue circles, lack of chelating quinolinium nitrogen by grey circles.

Characterization of employed cell line panel

Expression of Pgp in the applied cell line panel was confirmed by Westernblot. Furthermore, Expression of BCRP (ABC-G2) and MRP-1 (ABC-C1) was excluded by Westernblot. Functionality of Pgp is shown by Calcein-AM assay. The characterization of the panel is shown in Figure S2.

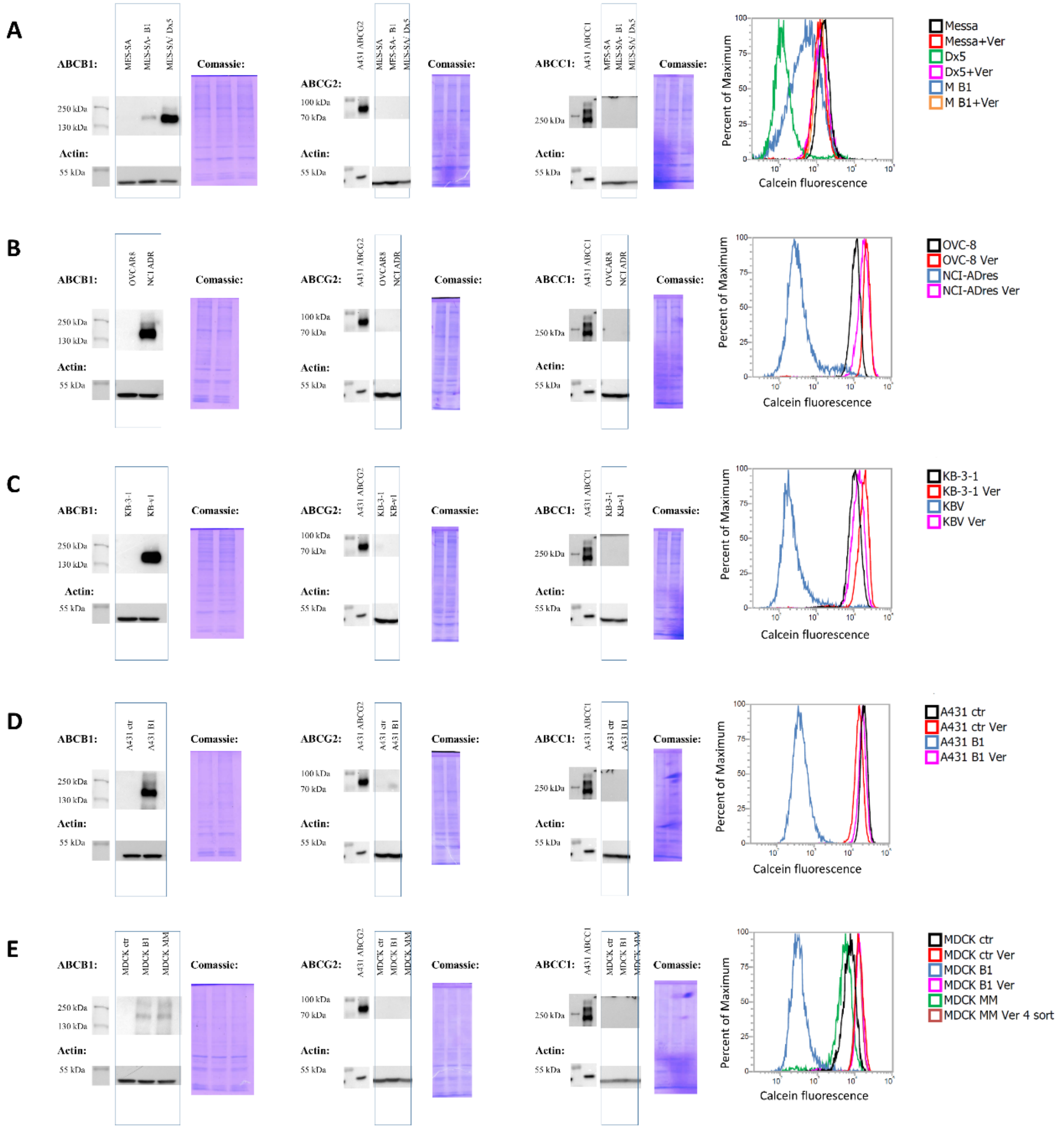

Figure S2: Characterization of the applied cell line panel. Panel (A): MES-SA, Dx5, B1, (B): OVCAR-8, NCI-ADRres, (C): KB-3-1, KB-v1, (D): A431, A431-B1, €: MDCK, MDCK-B1, MDCK-MM. Westernblots are shown for expression of ABC-B1, ABC-G2 and ABC-C1. Functionality of Pgp is confirmed by Calcein-AM assay. Histograms as indicated by colour code. Verapamil reverses reduced fluorescence.

Synthesis and Characterization of non-chelating derivatives

Compounds **NC-2, NC-3** and **NC-4** without chelating moieties were synthesized by the modified Mannich-reaction starting from 1-naphthol and the respective amines in the presence of paraformaldehyde. In case of **NC-4**, the ring-closed [1,3]naphthoxazine was obtained as an intermediate and cleaved under acidic conditions. Products were obtained in moderate to good yields.

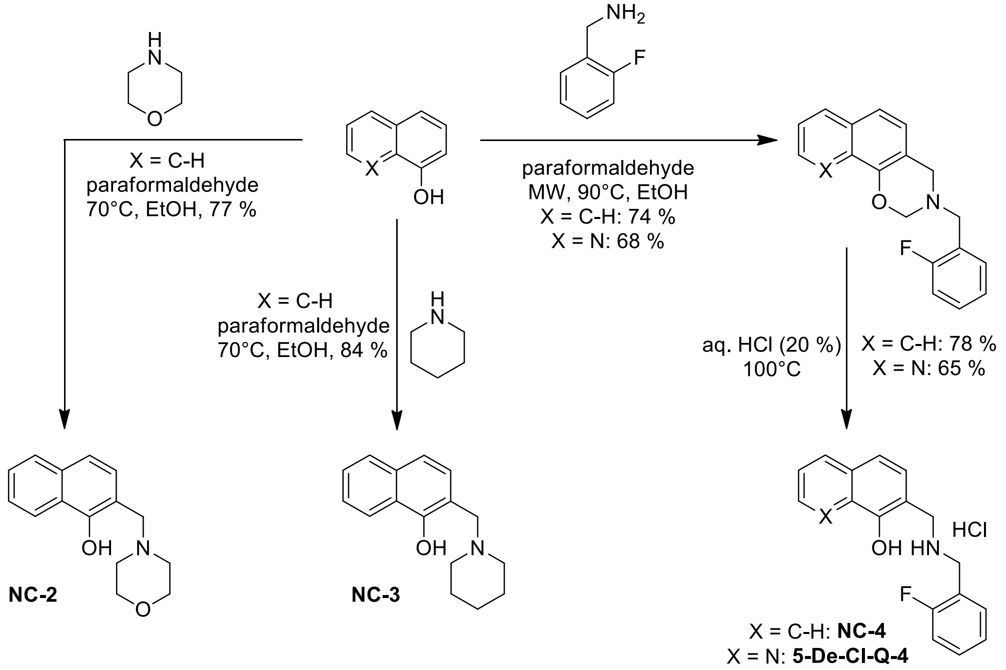

Scheme S1: Synthesis of the non-chelating derivatives 2-morpholinomethyl-1-naphthol (**NC-2**), 2-piperidin-1-yl-methyl-1-naphthol (**NC-3**) and 3-(2-fluorobenzyl)-naphth[2,1-*e*][1,3]oxazine (**NC-2**) and 2-(2-fluorobenzyl-aminomethyl-1-naphthol hydrochloride (**NC-4**) and chelating derivatives 3-(2-fluorobenzyl)-[1,3]oxazino[5,6-*h*]quinoline and 7-(2-fluorobenzyl-aminomethyl)-8-hydroxyquinoline hydrochloride (**5-De-Cl-Q-4**).

Instrumentation, detailed description and characterization of compounds

Chemicals used for synthesis were at least of reagent grade quality, obtained from commercial suppliers and used without further purification. Solvents were used as received or dried. All new compounds whose biological activity was evaluated in this work have a purity ≥98% as confirmed by NMR and/or elemental analysis.

^{1}H and ^{13}C NMR spectra were recorded in deuterated solvents on 600 MHz spectrometer at room temperature (^{1}H: 600 MHz, ^{13}C: 150 MHz). Chemical shifts δ are expressed in ppm values using the residual solvent peaks as internal standards ($CDCl_3$ 7.26; 77.16 ppm; DMSO-d_6 2.50; 39.52 ppm). Melting points were determined on a Hinotek X-4 type melting point apparatus and are uncorrected. Elemental analyses were performed with a Perkin-Elmer 2400 CHNS elemental analyzer. Merck Kieselgel $60F_{254}$ plates were used for TLC. Microwave reactions were performed by using CEM LabMate microwave reactor.

2-Morpholinomethyl-1-naphthol (NC-2).

1-Naphthol (0.40 g, 2.78 mmol), was reacted with morpholine (0.30 g, 3.44 mmol) and paraformaldehyde (0.208 g, 6.95 mmol) in ethanol (EtOH) (30 mL) for 8h at 70 ºC . The product was obtained as a beige oil in a yield of 77% (0.521g) after purification by column chromatography (eluent: *n*-hexane:EtOAc 2:1). ^{1}H NMR ($CDCl_3$, Figure S3): δ = 2.47-2.80 (4H, m); 3.77-3.85 (4H, m); 3.87 (2H, s); 7.10 (1H, d, J = 8.2 *Hz*); 7.33 (1H, d, J = 8.3 *Hz*); 7.45-7.51 (2H, m); 7.77 (1H, d, J = 8.4 *Hz*); 8.28 (1H, d, J = 8.2 *Hz*); ^{13}C NMR ($CDCl_3$, Figure S4): δ = 53.0; 62.1; 66.8; 113.1; 118.6; 122.0; 124.9; 126.1; 126.6; 127.3; 134.1; 153.3; Anal. calcd. for $C_{15}H_{17}NO_2$ (243.30): C, 74.05; H, 7.04; N, 5.76. Found: C, 74.27; H, 7.02; N, 5.72.

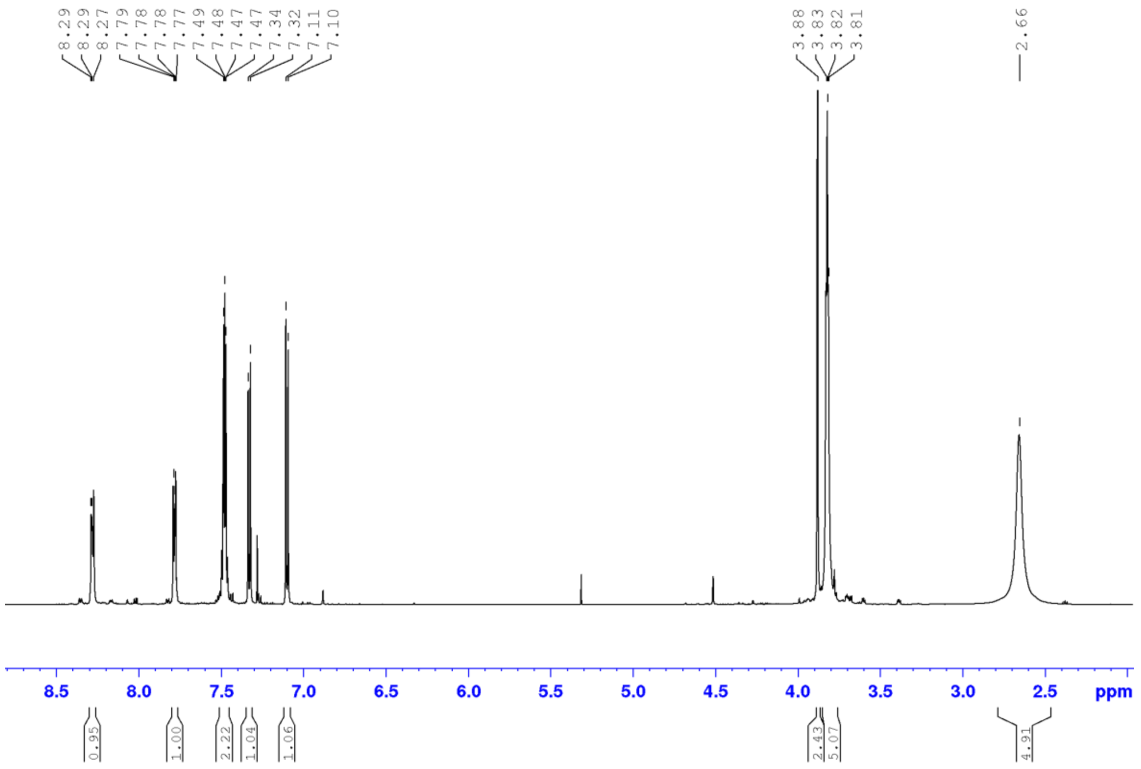

Figure S3: ^{1}H-NMR spectrum of **NC-2**

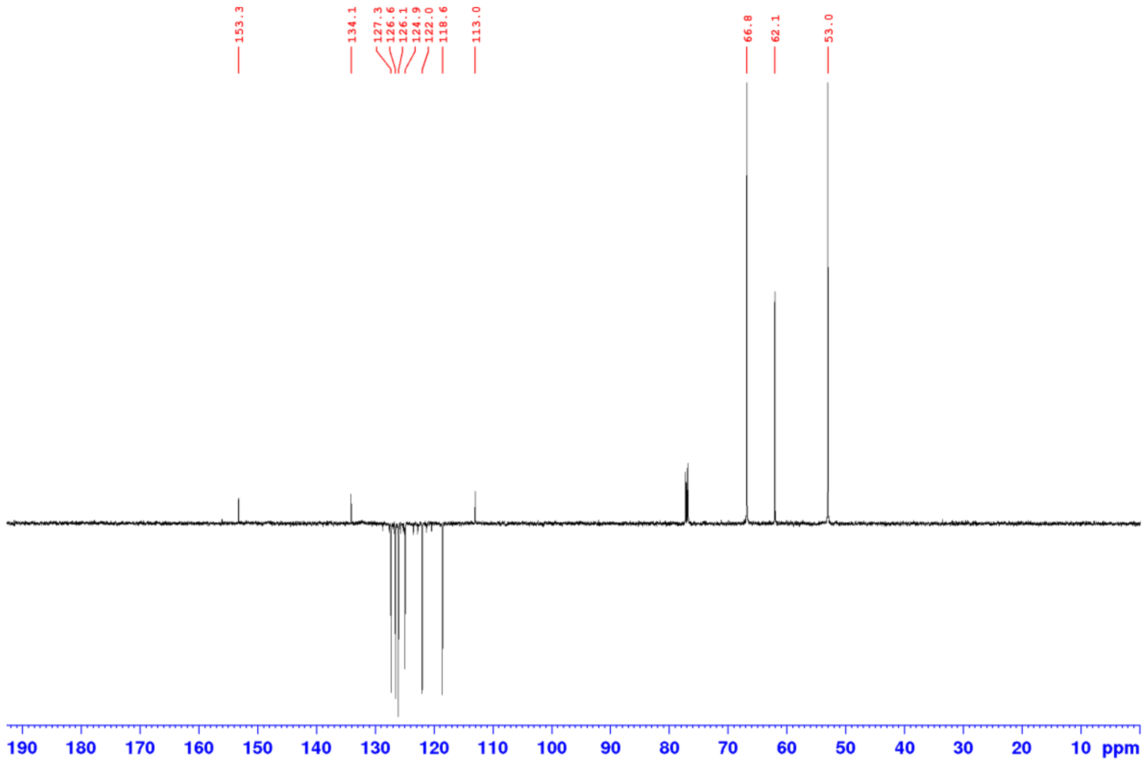

Figure S4: ^{13}C-NMR spectrum of **NC-2**

2-Piperidin-1-yl-methyl-1-naphthol (NC-3).

1-Naphthol (0.40 g, 2.78 mmol), was reacted with piperidine (0.30 g, 3.52 mmol) and paraformaldehyde (0.208 g, 6.95 mmol) in ethanol (EtOH) (30 mL) for 6h at 70 ºC for 8h. The product was purified by column chromatography (eluent: *n*-hexane:EtOAc 2:1) and isolated in a yield of 84% (0.563 g). ^{1}H NMR ($CDCl_3$, Figure S5): δ = 1.47-1.64 (2H, m); 1.67-1.74 (4H, m); 2.39-2.86 (4H, m); 3.85 (2H, s); 7.08 (1H, d, J = 8.3 *Hz*); 7.29 (1H, d, J = 8.1 *Hz*); 7.73-7.78 (2H, m); 8.27 (1H, d, J = 8.4 *Hz*); 8.11 (1H, d, J = 8.2 *Hz*); ^{13}C NMR ($CDCl_3$, Figure S6): δ = 24.2; 25.9; 54.0; 62.4; 113.7; 118.0; 122.1; 124.7; 125.0; 125.8; 126.5; 127.2; 133.9; 153.8; Mp.: 134-136 °C. Anal. calcd. for $C_{16}H_{19}NO$ (241.33): C, 79.63; H, 7.94; N, 5.80. Found: C, 79.51; H, 7.90; N, 5.82.

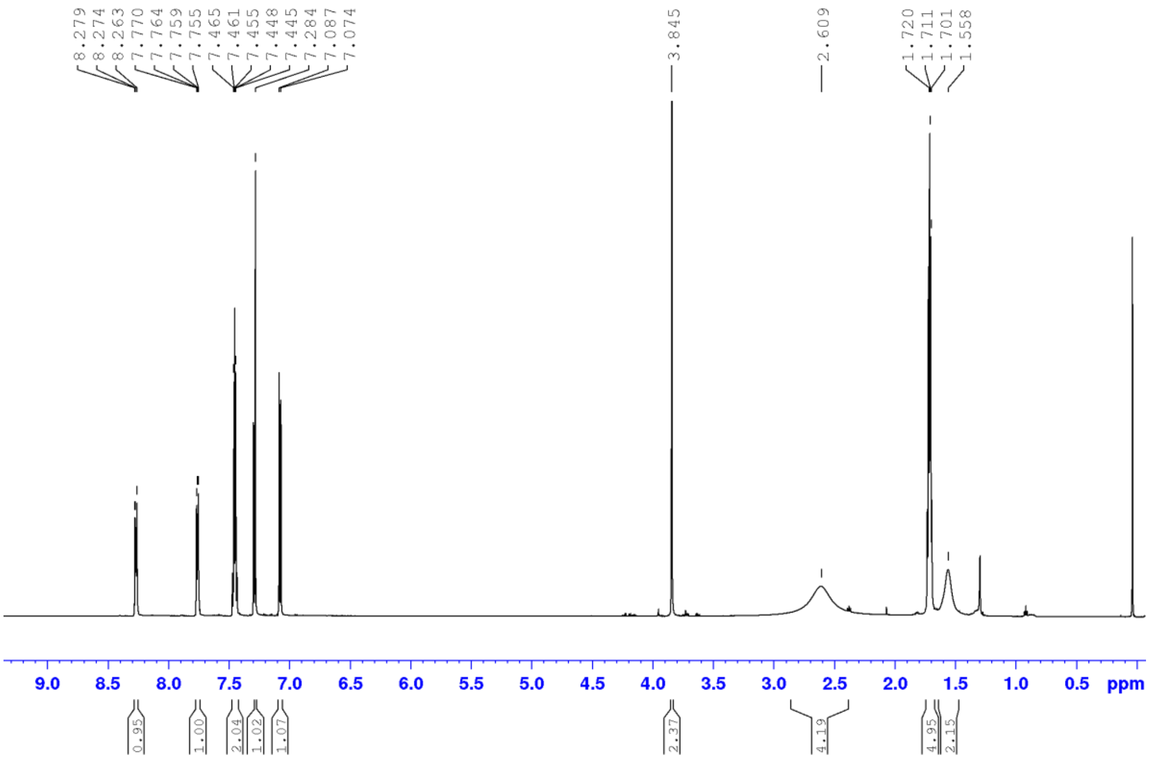

Figure S5: ^{1}H-NMR spectrum of **NC-3**

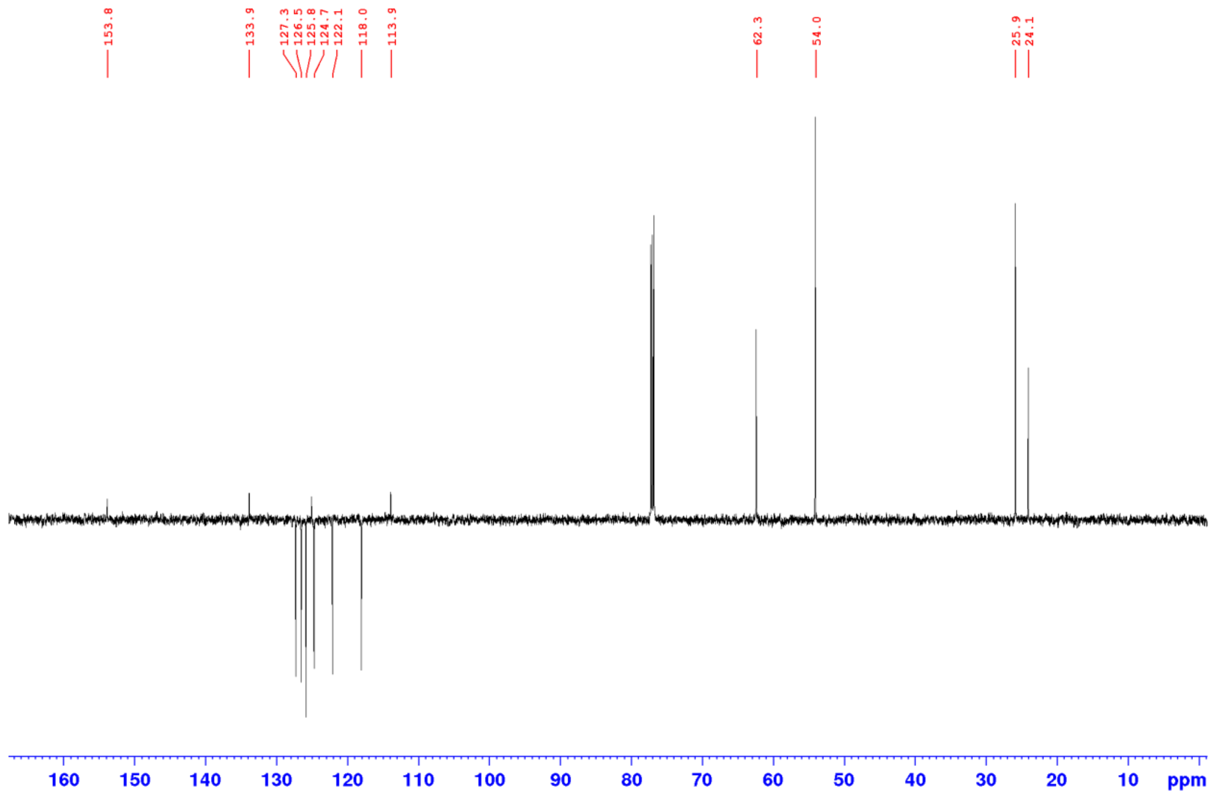

Figure S6: ^{13}C-NMR spectrum of **NC-3**

3-(2-Fluorobenzyl)-naphth[2,1-*e*][1,3]oxazine (intermediate).

1-naphthol (0.40 g, 2.78 mmol), 2-fluorobenzylamine (0.52 g, 4.15 mmol), paraformaldehyde (0.208 g, 6.95 mmol) and ethanol (EtOH) (20 mL) were placed in a 35 mL pressurized reaction vial. The mixture was heated by M.W irradiation at 100 ºC for 60 min. Upon evaporation of the solvent and crystallization from *n*-hexane:EtOAc (4:1; 25 mL), the product was obtained in a yield of 74% (0.603 g). ^{1}H NMR (DMSO-d_6, Figure S7): δ = 3.98 (2H, s); 4.05 (2H, s); 5.09 (2H, s); 7.12-7.25 (3H, m); 7.32-7.38 (1H, m); 7.41 (1H, d, J = 8.2 *Hz*); 7.45-7.51 (3H, m); 7.80-7.85 (1H, m); 8.03-8.08 (1H, m); ^{13}C NMR (DMSO-d_6, Figure S8): δ = 48.7; 49.6; 83.1; 114.4; 115.7 ($^2J_{C-F}$ = 21.8 *Hz*); 120.0; 121.2; 124.6; 124.9; 125.6 ($^2J_{C-F}$ = 14.4 *Hz*); 126.3 ($^3J_{C-F}$ = 10.3 *Hz*); 128.0; 129.8 ($^3J_{C-F}$ = 8.5 *Hz*); 131.5; 133.4; 149.0; 161.2 ($^1J_{C-F}$ = 245.5 *Hz*). Mp.: 85-87 °C. Anal. calcd. for $C_{19}H_{16}FNO$ (293.33): C, 77.80; H, 5.50; N, 4.77. Found: C, 77.61; H, 5.48; N, 4.75.

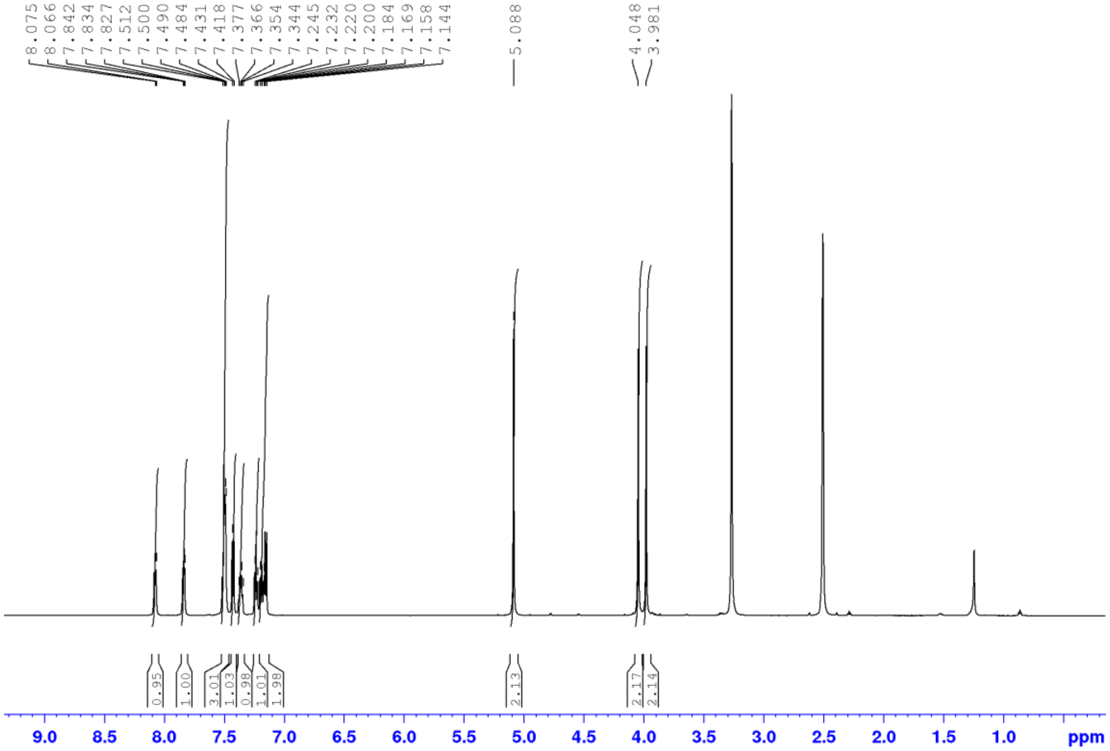

Figure S7: ^{1}H-NMR spectrum of 3-(2-Fluorobenzyl)-naphth[2,1-*e*][1,3]oxazine

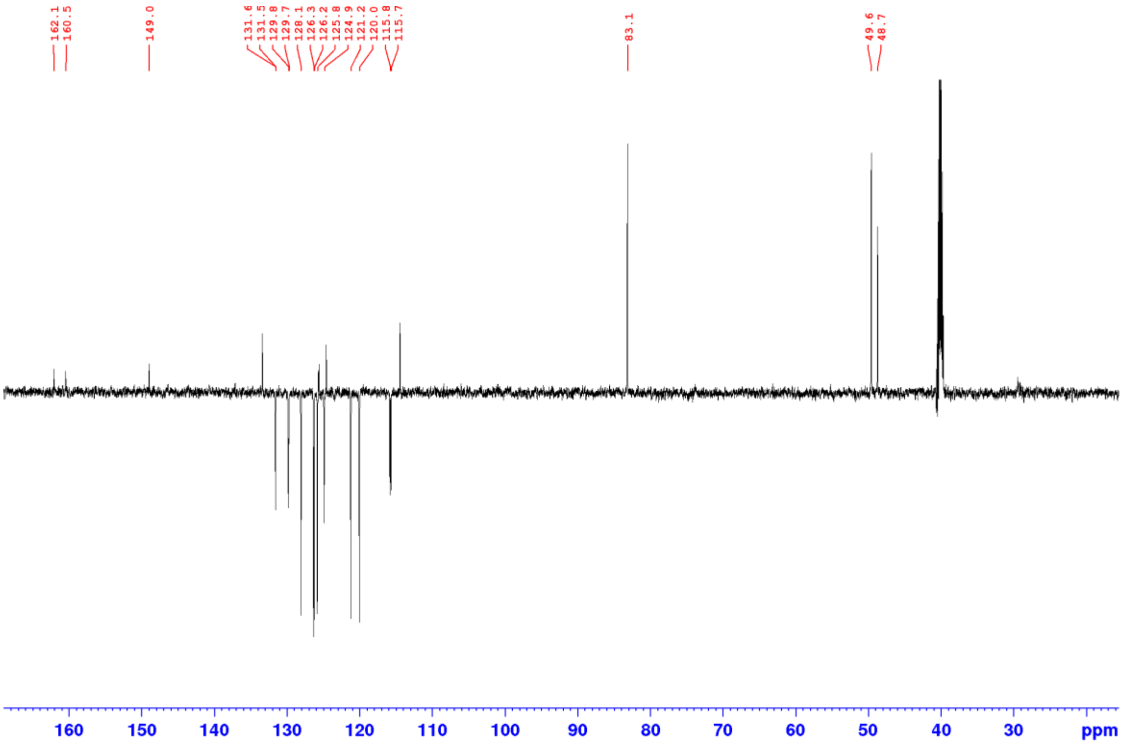

Figure S8: ^{13}C-NMR spectrum of 3-(2-Fluorobenzyl)-naphth[2,1-*e*][1,3]oxazine

2-(2-Fluorobenzyl-aminomethyl-1-naphthol hydrochloride (NC-4).

Hydrolytic cleavage of the intermediate (0.3 g, 1.02 mmol) was performed by refluxing a suspension in 70 mL aq. HCl (20 %) for 2.5 h. Upon evaporation of the solvent, the crude product was crystallized from EtOAc (25 mL) and recrystallized with EtOH (30 mL). The final product was obtained in a yield of 78% (0.253 g);. ^{1}H NMR (DMSO-d_6, Figure S9): δ = 4.14 (2H, s); 4.27 (2H, s); 7.16-7.34 (3H, m); 7.39-7.57 (3H, m); 7.64 (1H, t, J = 8.1 *Hz*); 8.06 (1H, d, J = 8.3 *Hz*); 8.37 (1H, d, J = 8.1 *Hz*); 9.50 (2H, brs); 9.91 (1H, brs); ^{13}C NMR (DMSO-d_6, Figure S10): δ = 43.2; 46.1; 113.0; 115.9 ($^{2}J_{C\text{-}F}$ = 21.8 *Hz*); 119.5 ($^{2}J_{C\text{-}F}$ = 13.7 *Hz*); 123.7; 124.4; 124.9; 125.5; 126.5; 127.5; 128.2; 130.4; 131.8 ($^{3}J_{C\text{-}F}$ = 8.1 *Hz*); 132.8 133.5; 151.3; 161.1 ($^{1}J_{C\text{-}F}$ = 250.9 *Hz*). Mp.: >320 °C. Anal. calcd. for $C_{18}H_{17}ClFNO$ (317.79): C, 68.03; H, 5.39; N, 4.41. Found: C, 67.81; H, 5.42; N, 4.39.

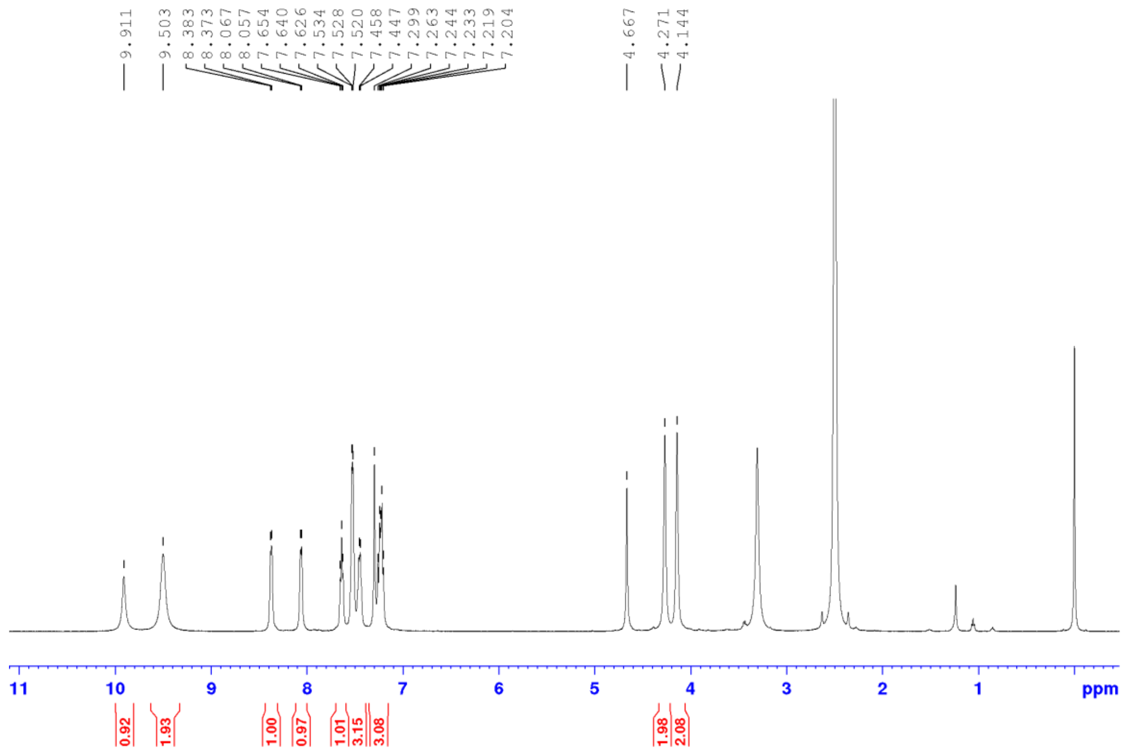

Figure S9: ^{1}H-NMR spectrum of **NC-4**

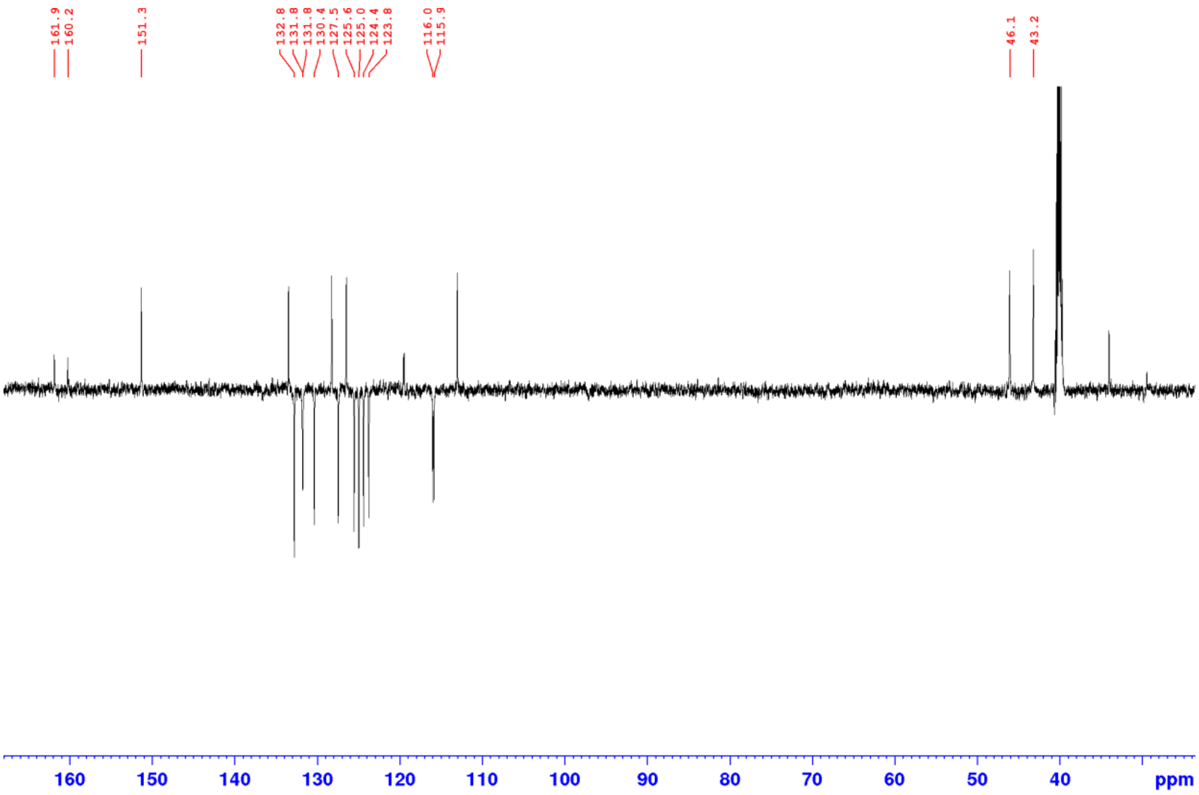

Figure S10: ^{13}C-NMR spectrum of **NC-4**

3-(2-fluorobenzyl)-[1,3]oxazino[5,6-*h*]quinoline (intermediate).

8-Hydroxyquinoline (0.50 g, 3.44 mmol), 2-fluorobenzylamine (0.64 g, 5.16 mmol), paraformaldehyde (0.312 g, 10.42 mmol) and ethanol (EtOH) (20 mL) were placed in a 35 mL pressurized reaction vial. The mixture was heated by M.W irradiation at 100 ºC for 90 min. Upon evaporation of the solvent and crystallization from Et_2O (20 mL), the product was obtained in a yield of 68% (0.688 g). ^{1}H NMR (DMSO-d_6, Figure S11): δ = 3.98 (2H, s); 4.09 (2H, s); 5.08 (2H, s); 7.15-7.27 (3H, m); 7.33-7.40 (1H, m); 7.44 (1H, d, J = 8.1 *Hz*); 7.47-7.54 (2H, m); 8.26 (1H, d, J = 8.2 *Hz*); 8.83 (1H, s); ^{13}C NMR (DMSO-d_6, Figure S12): δ = 48.8; 49.8; 82.9; 115.7 ($^{2}J_{C-F}$ = 18.2 *Hz*); 118.4; 119.3; 121.8; 124.9; 125.6 ($^{2}J_{C-F}$ = 13.6 *Hz*); 126.8; 128.2; 129.8 ($^{3}J_{C-F}$ = 8.3 *Hz*); 131.6 ($^{3}J_{C-F}$ = 5.1 *Hz*); 136.2; 139.3; 149.5; 149.6; 161.2 ($^{1}J_{C-F}$ = 244.6 *Hz*). Mp.: 113-115 °C. Anal. calcd. for $C_{18}H_{15}FN_2O$ (294.32): C, 73.45; H, 5.14; N, 9.52. Found: C, 74.02; H, 5.18; N, 9.48.

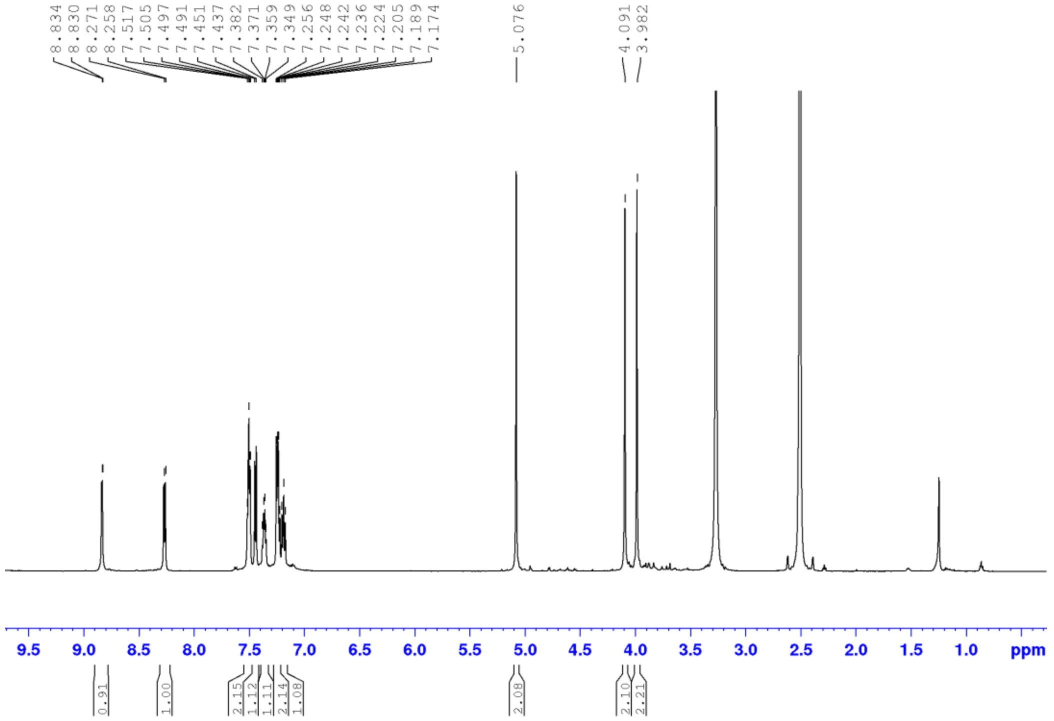

Figure S11: ^{1}H-NMR spectrum of 3-(2-fluorobenzyl)-[1,3]oxazino[5,6-*h*]quinoline

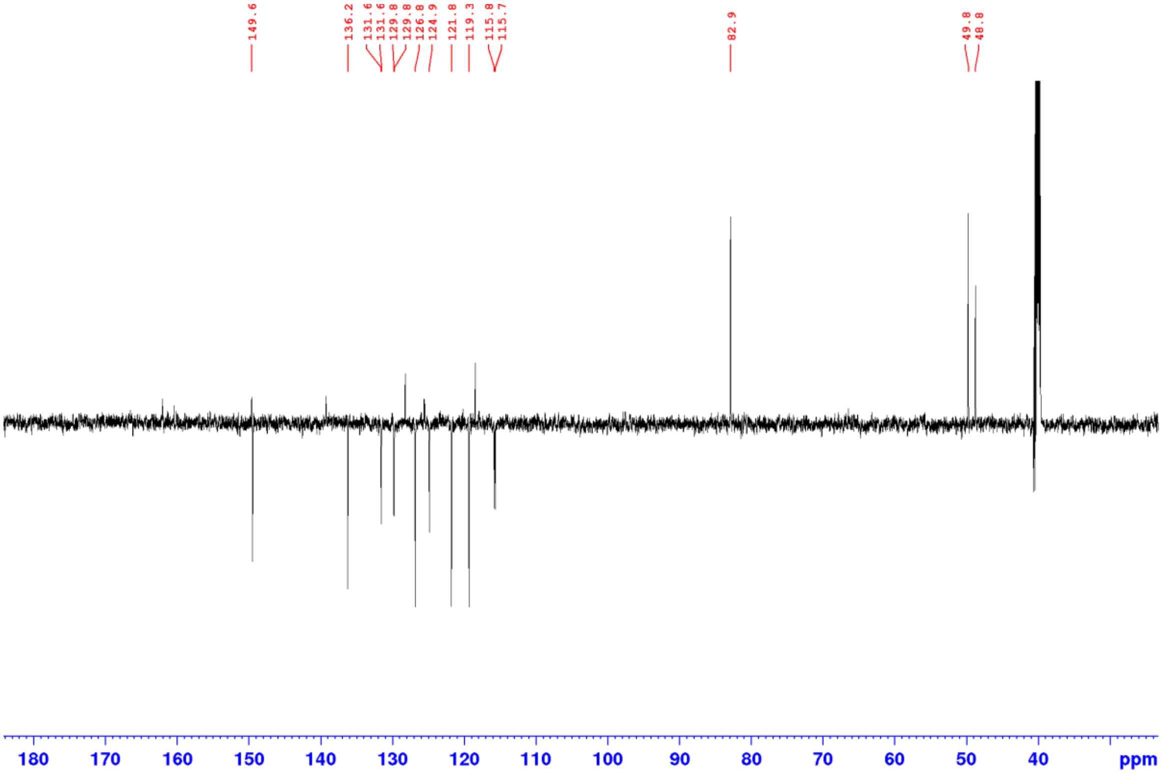

Figure S12: ^{13}C-NMR spectrum of 3-(2-fluorobenzyl)-[1,3]oxazino[5,6-*h*]quinoline

7-(2-fluorobenzyl-aminomethyl)-8-hydroxyquinoline hydrochloride (5-De-Cl-Q-4).

Hydrolytic cleavage of the intermediate (0.5 g, 1.70 mmol) was performed by refluxing a suspension in 70 mL aq. HCl (20 %) for 5.0 h. Upon evaporation of the solvent, the crude product was crystallized from EtOAc (30 mL) and recrystallized with EtOH: Et_2O (3:1; 30 mL). The final product was obtained in a yield of 65% (0.352 g);. ^{1}H NMR (DMSO-*d*$_6$, Figure S13): δ = 4.27 (2H, s); 4.45 (2H, s); 7.23-7.30 (2H, m); 7.44-7.50 (2H, m); 7.60 (1H, d, J = 8.2 *Hz*); 7.74-7.81 (2H, m); 7.86 (1H, d, J = 8.1 *Hz*); 8.62 (1H, d, J = 8.0 *Hz*); 9.00 (1H, s); 9.89 (2H, brs); ^{13}C NMR (DMSO-*d*$_6$, Figure S14): δ = 43.4; 45.2; 116.0 ($^{2}J_{C\text{-}F}$ = 21.3 *Hz*); 118.5; 119.6 ($^{2}J_{C\text{-}F}$ = 14.8 *Hz*); 125.0 ($^{3}J_{C\text{-}F}$ = 3.8 *Hz*); 129.6; 130.9; 131.8 ($^{3}J_{C\text{-}F}$ = 8.6 *Hz*); 132.9; 148.0; 151.3; 161.2 ($^{1}J_{C\text{-}F}$ = 243.4 *Hz*). Mp.: 179-181 °C. Anal. calcd. for $C_{17}H_{16}ClFN_2O$ (318.77): C, 64.05; H, 5.06; N, 8.79. Found: C, 64.18; H, 5.09; N, 8.81.

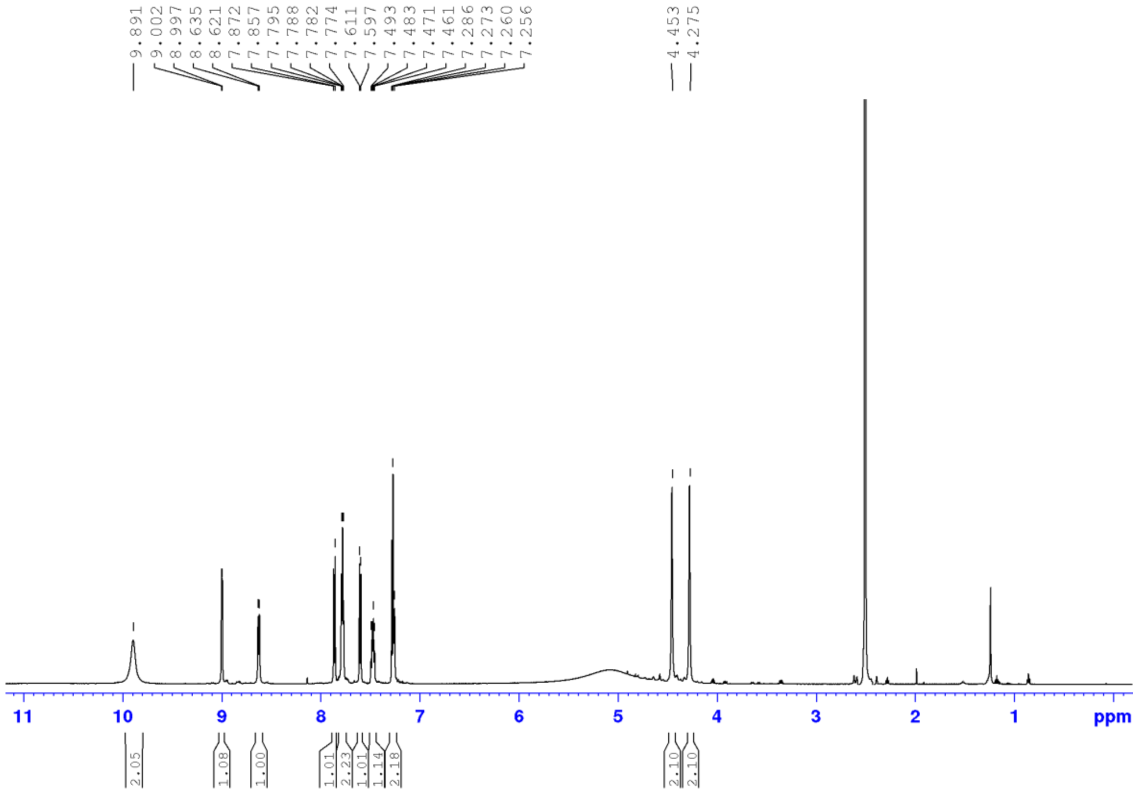

Figure S13: ^{1}H-NMR spectrum of **5-De-Cl-Q-4**

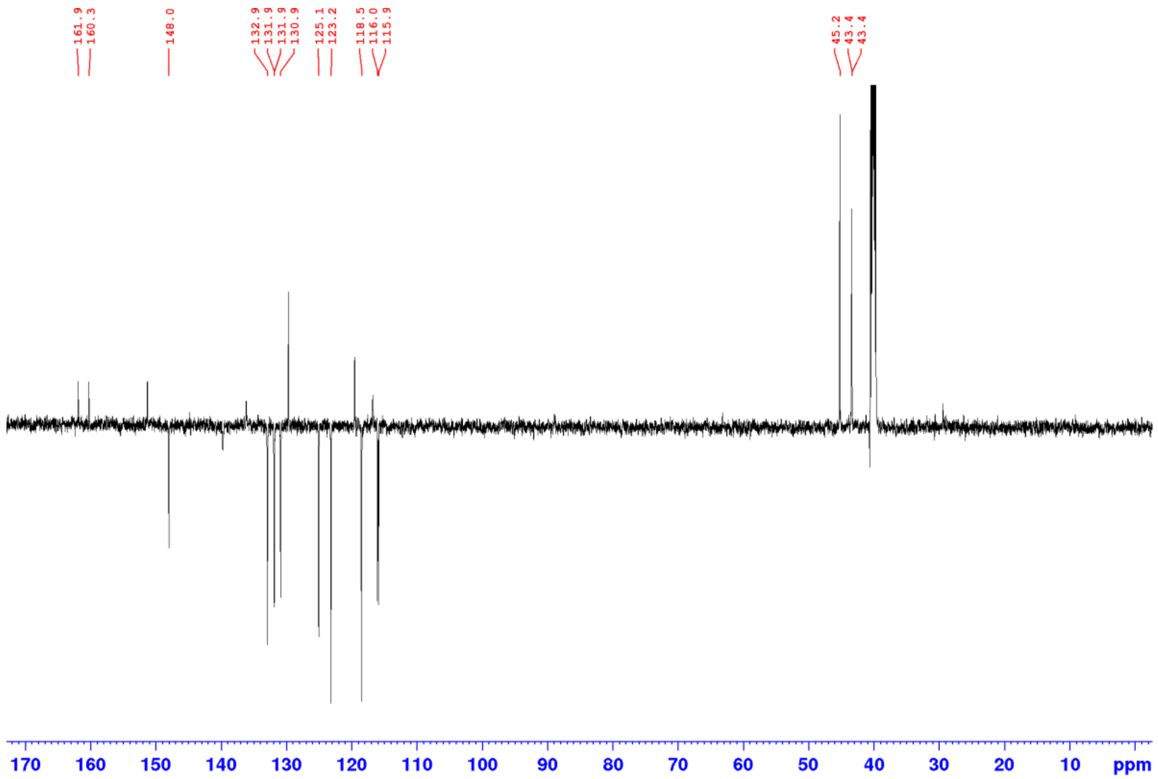

Figure S14: ^{13}C-NMR spectrum of **5-De-Cl-Q-4**

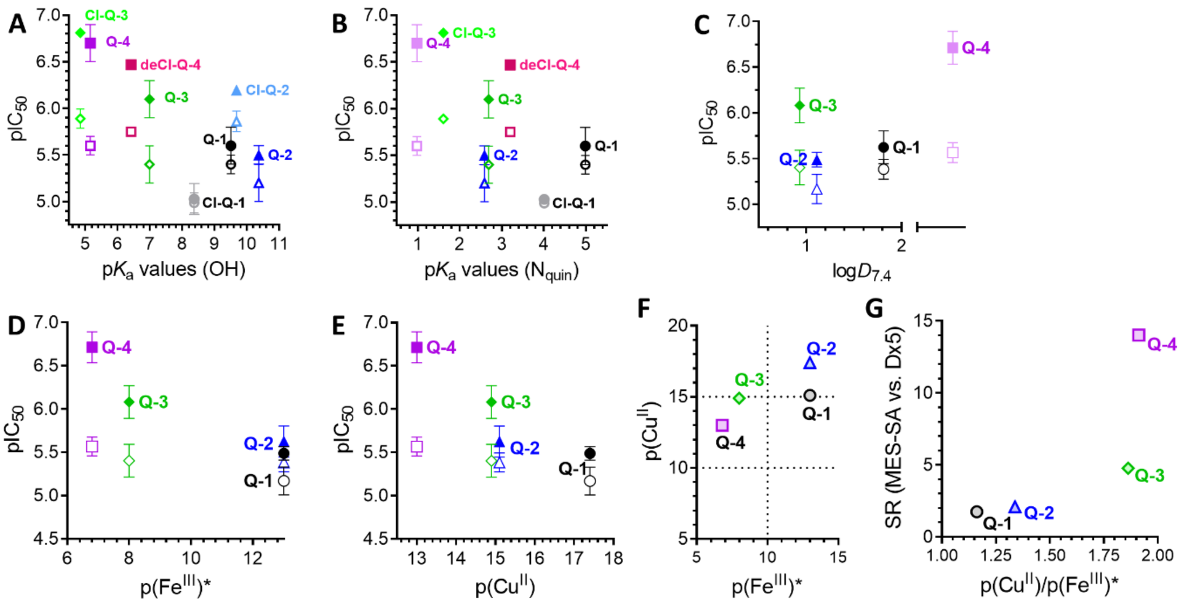

Figure S15: pK_a values influence MDR-selective toxicity of 8-hydroxyquinoline derivatives. Toxicity (pIC_{50}) values shown Table X were plotted against the phenolic-OH pK_a (A) and quinolinium nitrogen pK_a (B) of **Q-1** (black circles), **Q-2** (blue triangles), **Q-3** (green diamonds) and **Q-4** (purple squares) (57), as well as for **Cl-Q-1** (grey circles), **Cl-Q-2** (light blue triangles), **Cl-Q-3** (light green diamonds) and **de-Cl-Q-4** (magenta squares). Even though pK_a values have an impact on lipophilicity (log$D_{7.4}$ values), there is no comparable relation between toxicity and log$D_{7.4}$ values (C). In contrast, iron(III) (D) and copper(II) (E) binding capacities of the ligands, expressed as pM-values ($p(Cu^{II}) = -\log[Cu^{II}]$, $p(Fe^{III})^* = -\log([Fe^{III}] + i\times\Sigma[Fe_i(OH)_j])$, both calculated at pH = 7.4, with c_M= 1 μM; c_L= 10 μM) have an impact on the MDR-selective cytotoxicity of the ligands. (F) Relative iron(III) and copper(II) binding ability of ligands **Q-1** to **Q-4** (expressed as pM* values). (G). Relation of preferential copper binding to MDR-selective toxicity (SR). The quinolinium nitrogen pK_a value and the log$D_{7.4}$ value for **Q-4** were not reliably measureable and were hence predicted using ChemAxon tools. Predicted values are highlighted with pale color.

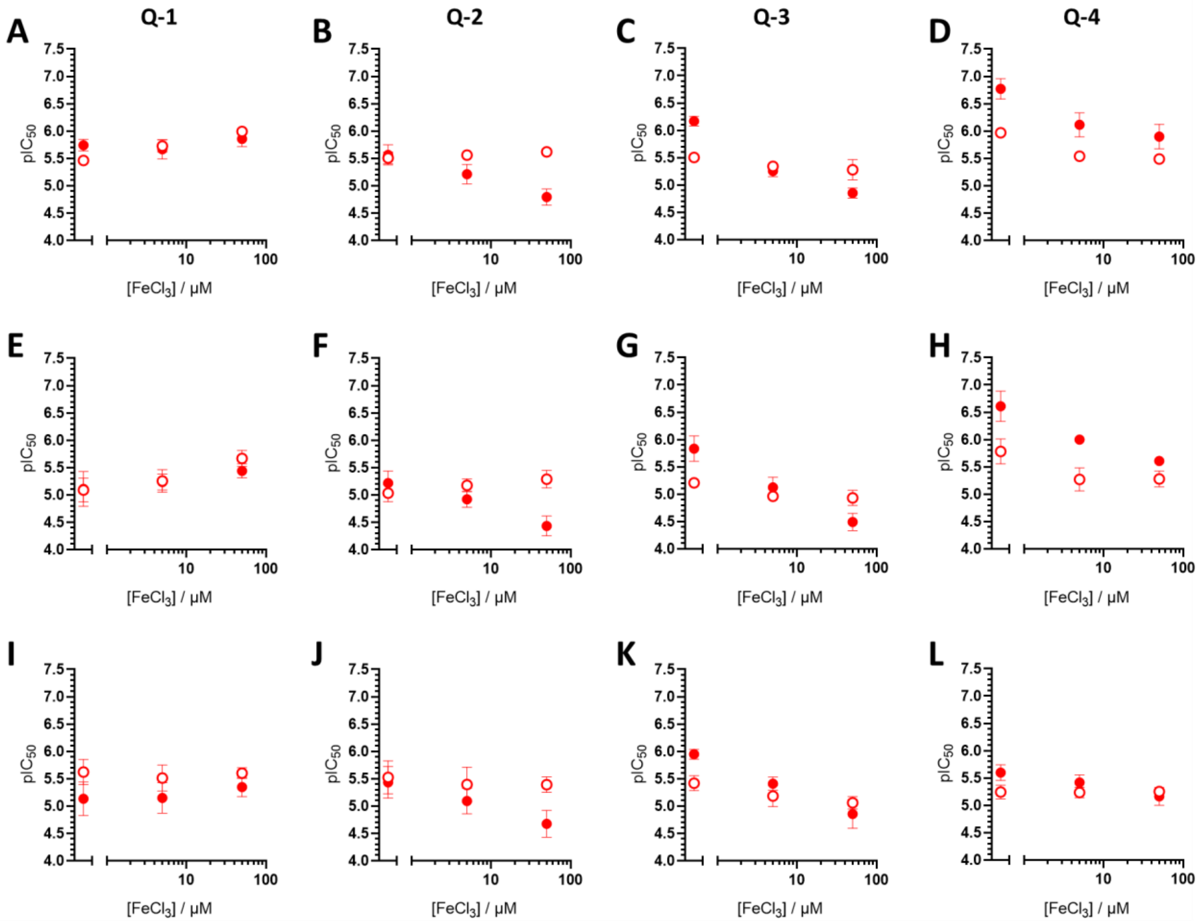

Figure S16: Effect of increasing concentrations of $FeCl_3$ co-administration on toxicity of the ligands **Q-1** (A,E,I), **Q-2** (B,F.J), **Q-3** (C,G,K) and **Q-4** (D,H,L) on further cell line pairs. pIC_{50} values of the ligands in presence of indicated concentrations of $FeCl_3$were obtained after 72 h incubation of parental (empty symbols, panels A-D: OVCAR-8, panels E-H: KB-3-1, panels I-L: A431) and P-gp expressing (filled symbols, panels A-D: NCI-adrRes, panels E-H: KB-v1, panels I-L: A431-B1) cells. Data represent mean values and standard deviations obtained in at least three independent experiments.

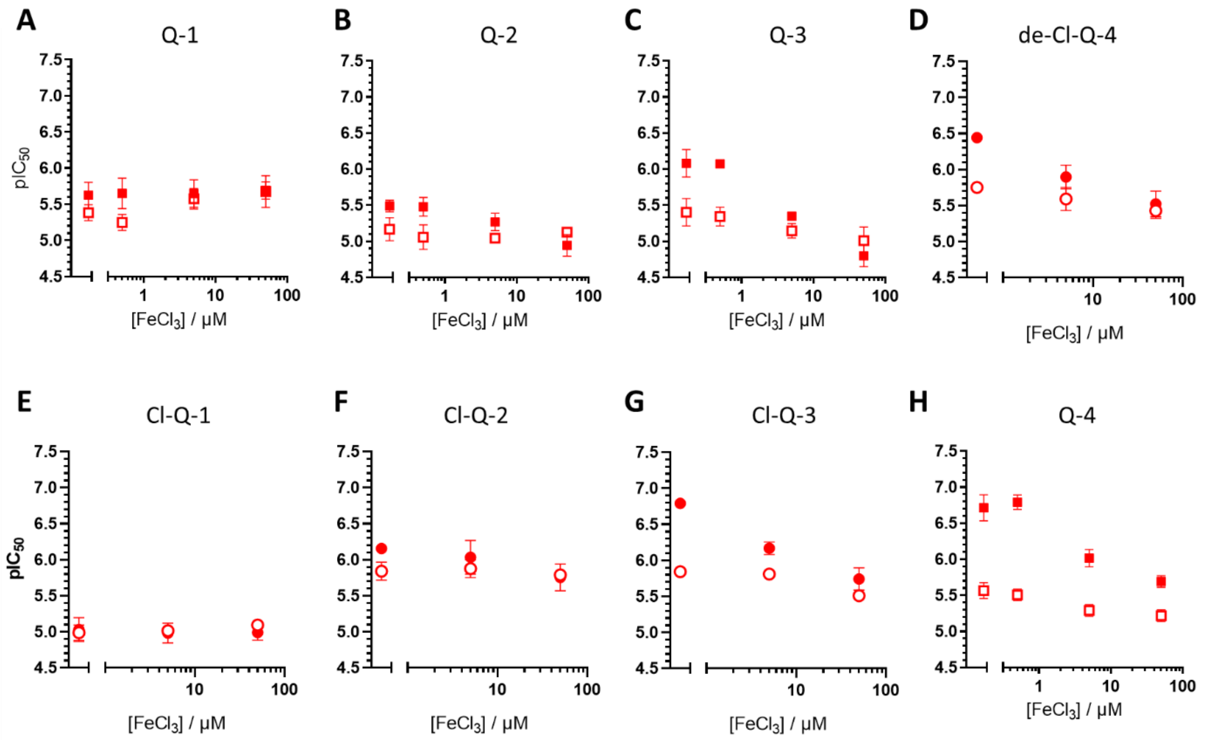

Figure S17: Effect of increasing concentrations of $FeCl_3$ co-administration on toxicity of the (R5-) un-chlorinated ligands: **Q-1** (A), **Q-2** (B), **Q-3** (C) and **de-Cl-Q-4** (D) as well as of ligands with chloro-substituent in R5: **Cl-Q-1** (E), **Cl-Q-2** (F), **Cl-Q-3** (G) and **Q-4** (H). pIC_{50} values of the ligands in presence of indicated concentrations of $FeCl_3$ were obtained after 72 h incubation of parental MES-SA (empty symbols) and P-gp expressing MES-SA/Dx5 cells (filled symbols). Data represent mean values and standard deviations obtained in at least three independent experiments.

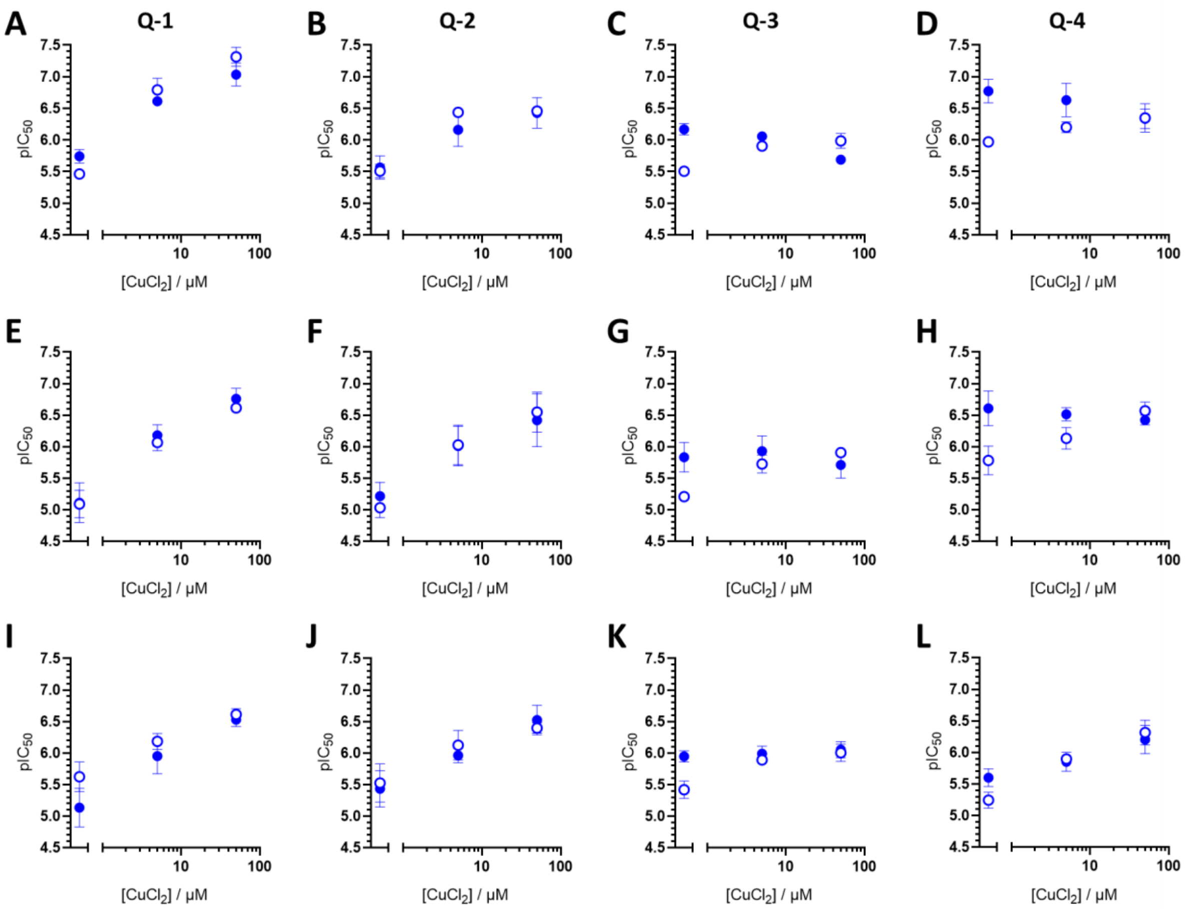

Figure S18: Effect of increasing concentrations of $CuCl_2$ co-administration on toxicity of the ligands **Q-1** (A,E,I), **Q-2** (B,F.J), **Q-3** (C,G,K) and **Q-4** (D,H,L) on further cell lines. pIC_{50} values of the ligands in presence of indicated concentrations of $CuCl_2$ were obtained after 72 h incubation of parental (empty symbols, panels A-D: OVCAR-8, panels E-H: KB-3-1, panels I-L: A431) and Pgp expressing (filled symbols, panels A-D: NCI-adrRes, panels E-H: KB-v1, panels I-L: A431-B1) cells. Data represent mean values and standard deviations obtained in at least three independent experiments.

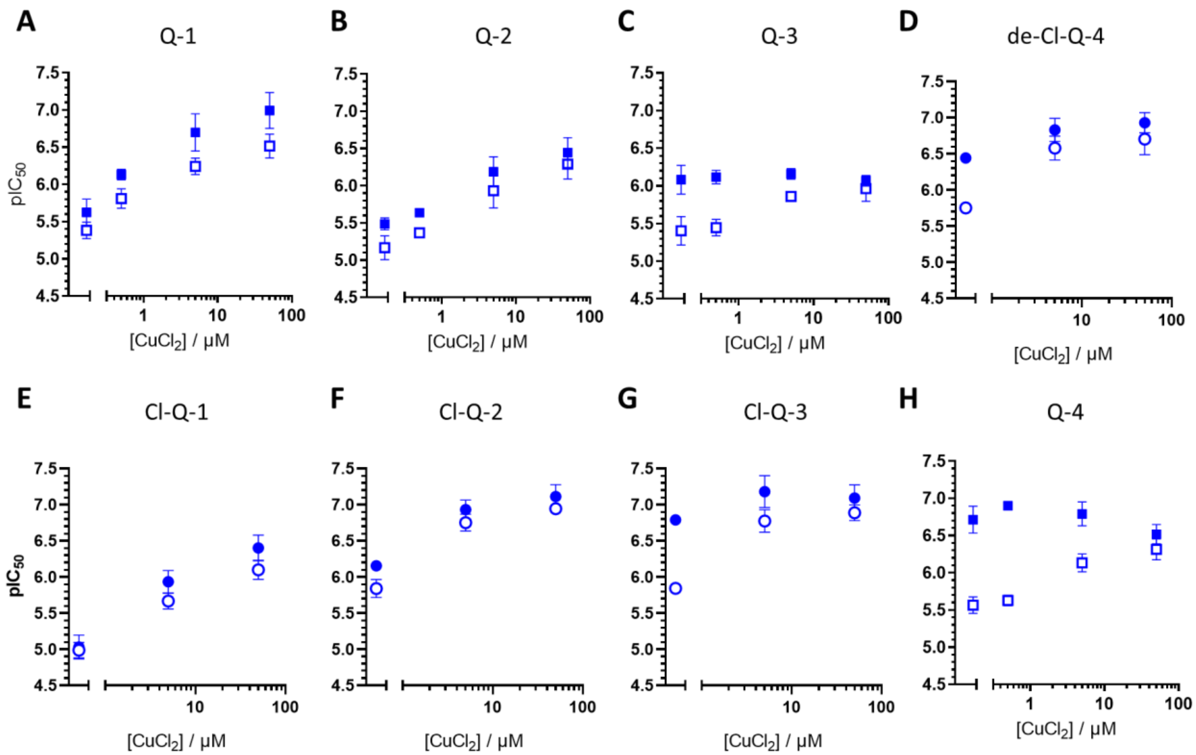

Figure S19: Effect of increasing concentrations of $CuCl_2$ co-administration on toxicity of the (R5-) un-chlorinated ligands: **Q-1** (A), **Q-2** (B), **Q-3** (C) and **de-Cl-Q-4** (D) as well as of ligands with chloro-substituent in R5: **Cl-Q-1** (E), **Cl-Q-2** (F), **Cl-Q-3** (G) and **Q-4** (H). pIC_{50} values of the ligands in presence of indicated concentrations of $CuCl_2$ were obtained after 72 h incubation of parental MES-SA (empty symbols) and P-gp expressing MES-SA/Dx5 cells (filled symbols). Data represent mean values and standard deviations obtained in at least three independent experiments.

Table S1: Toxicity of ligands and *in situ* preformed complexes in MES-SA and MES-SA/Dx5 cells in the absence and presence of 1 μM TQ. IC_{50} values and standard deviation of at least three independent experiments are given in μM calculated as ligand equivalents. Selectivity-ratios are given as the ratio of IC_{50} values obtained in Pgp negative divided by the Pgp positive cells. In order to avoid misinterpretation due to different amount of ligand present in the respective complexes, concentrations are expressed as ligand equivalents. (Therefore at the 1:3 metal-to-ligand ratio, the IC_{50} values refer to "1/3 [ML_3]".) While complexation with iron(III) seems to slightly decrease toxicity of the ligand, complexation with copper(II) increases the efficiency. Even though with pre-formed complexes similar trends could be observed as in the co-incubation experiments, these trends were much less pronounced in case of the pre-mixed complexes.

M:L ratio		MES-SA	Dx5	SR	MES-SA_TQ	Dx5_TQ	SR_TQ
	**Q-1**	4.19±0.74	2.46±0.69	1.70	5.08±0.48	2.94±0.66	1.73
1:3	1/3 [Fe(**Q-1**)$_3$]	3.31±0.52	2.92±0.80	1.13	3.07±0.36	2.96±0.64	1.04
1:2	1/2 [Fe(**Q-1**)$_2$]	5.43±2.81	4.16±1.40	1.31	3.43±0.66	2.88±0.62	1.19
1:1	[Fe(**Q-1**)]	3.00±0.46	2.76±0.70	1.09	3.50±0.74	3.03±0.64	1.16
1:2	1/2 [Cu(**Q-1**)$_2$]	3.77±0.66	2.03±0.46	1.86	4.14±1.20	2.61±0.44	1.58
1:1	[Cu(**Q-1**)]	1.36±0.25	1.09±0.17	1.25	1.81±0.32	1.16±0.11	1.57
	**Q-2**	6.97±1.75	3.27±0.39	2.13	4.65±0.92	5.49±1.02	0.85
1:3	1/3 [Fe(**Q-2**)$_3$]	3.87±0.88	3.13±0.84	1.24	4.35±0.58	4.23±0.52	1.03
1:2	1/2 [Fe(**Q-2**)$_2$]	3.60±0.57	4.22±1.67	0.85	3.90±0.57	3.72±0.54	1.05
1:1	[Fe(**Q-2**)]	5.24±0.96	8.37±2.28	0.63	4.22±0.26	3.46±0.64	1.22
1:2	1/2 [Cu(**Q-2**)$_2$]	2.27±0.67	1.76±0.56	1.29	2.02±0.65	1.92±0.45	1.05
1:1	[Cu(**Q-2**)]	1.54±0.55	1.28±0.29	1.20	2.03±1.13	1.76±0.49	1.15
	**Q-3**	4.11±1.20	0.86±0.26	4.75	2.99±0.91	3.15±0.93	0.95
1:3	1/3 [Fe(**Q-3**)$_3$]	2.12±0.68	0.91±0.22	2.33	1.82±0.71	1.45±0.25	1.25
1:2	1/2 [Fe(**Q-3**)$_2$]	5.52±2.63	1.70±0.57	3.24	6.49±1.35	4.18±0.89	1.55
1:1	[Fe(**Q-3**)]	5.20±1.04	2.08±0.82	2.50	4.42±0.91	4.47±0.99	0.99
1:2	1/2 [Cu(**Q-3**)$_2$]	1.91±0.59	0.76±0.26	2.52	2.13±0.65	2.04±0.36	1.04
1:1	[Cu(**Q-3**)]	1.95±0.55	0.77±0.14	2.54	2.02±1.01	1.36±0.25	1.49
	**Q-4**	2.75±0.46	0.20±0.06	13.68	2.22±0.36	3.28±0.51	0.68
1:3	1/3 [Fe(**Q-4**)$_3$]	2.95±1.33	0.22±0.12	13.14	3.54±0.13	3.45±0.33	1.03
1:2	1/2 [Fe(**Q-4**)$_2$]	4.59±1.18	0.30±0.08	15.07	4.23±0.81	4.38±0.86	0.97
1:1	[Fe(**Q-4**)]	7.17±1.82	0.33±0.06	21.51	5.12±0.55	5.20±0.44	0.98
1:2	1/2 [Cu(**Q-4**)$_2$]	1.46±0.16	0.29±0.039	5.03	1.36±0.26	1.22±0.025	1.11
1:1	[Cu(**Q-4**)]	1.04±0.12	0.27±0.024	3.86	1.00±0.019	0.89±0.084	1.12

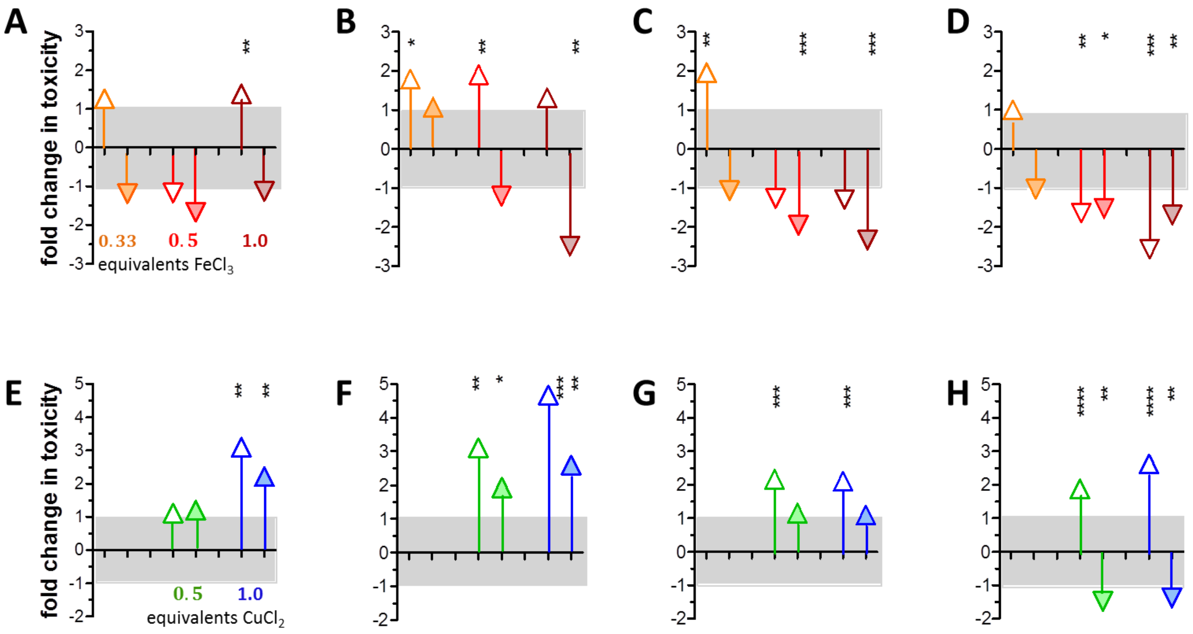

Figure S20: Fold change in toxicity of ligands **Q-1** (A, E), **Q-2** (B, F), **Q-3** (C, G) and **Q-4** (D, H) upon complexation with 0.33 (orange), 0.5 (red), or 1 (bordeaux) equivalents of $FeCl_3$ (A-D), or with 0.5 (green), or 1 (blue) equivalents of $CuCl_2$ (E-H). Changes in toxicity against MES-SA (open arrowheads) and MES-SA/Dx5 (filled arrowheads) cells were calculated by comparison of the average IC_{50} values of at least three independent experiments. Statistical significance of the difference toward the free ligands' behavior was calculated with t-test (*: $p \leq 0.05$, **: $p \leq 0.01$, ***: $p \leq 0.001$, ****: $p \leq 0.0001$).

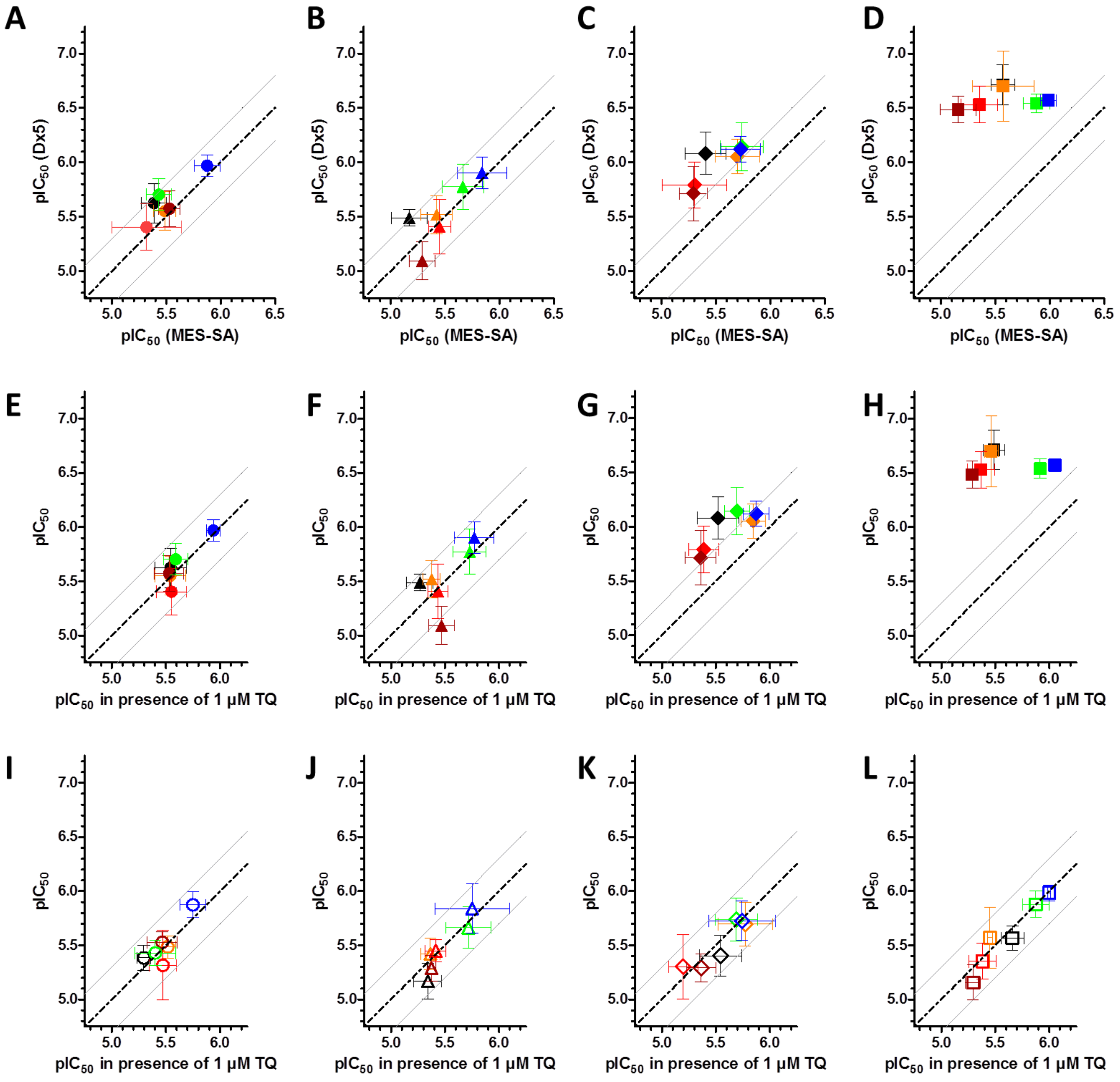

Figure S21: Impact of Pgp on toxicity of ligands (black, **Q-1** (A), **Q-2** (B), **Q-3** (C) and **Q-4** (D)) and complexes at different metal-to-ligand ratios illustrated by different colors: Fe:L = 1:3 (orange), 1:2 (light red), 1:1 (bordeaux), Cu:L = 1:2 (green), 1:1 (blue) and L (black). The toxic activity (pIC_{50} values) is compared in Pgp negative (MES-SA, x-axis) and Pgp positive (Dx5, y-axis) cells (A-D). While having no effect on the toxicity of ligand and complexes of **Q-1** (E) and **Q-2** (F), the Pgp inhibitor TQ decreases the activity of MDR selective ligands and complexes (**Q-3** (G), **Q-4** (H)). Expectedly, toxicity in Pgp negative MES-SA cells is not altered by co-administration of TQ (black, **Q-1** (I), **Q-2** (J), **Q-3** (K) and **Q-4** (L)).

Table S2: Effect of co-administered NAC, referring to Figure 7. Data represent average IC_{50} values in μM of at least three independent experiments with standard deviations.

Cell line	**MES-SA**	**MES-SA + NAC**	**Dx5**	**Dx5 + NAC**
**Q-1**	4.19 ±0.74	16.50 ±1.89	2.46 ±0.69	10.96 ±0.73
**Q-2**	6.97 ±1.75	18.83 ±4.37	3.27 ±0.39	4.67 ±0.61
**Q-3**	4.11 ±1.20	4.48 ±1.23	0.86 ±0.26	0.83 ±0.20
**Q-4**	2.75 ±0.46	2.19 ±0.86	0.20 ±0.06	0.23 ±0.06

Table S3: Characterization of intracellular ROS production with the DCFDA assay for P-gp negative MES-SA and P-gp positive MES-SA/Dx5 cells. Fold changes in fluorescence are shown after a 2 h with the investigated 8-hydroxyquinoline ligands. Data S-refer to Figure 7 and allow additional comparison of R5 unsubstituted and chloro-substituted derivatives of **Q-1** to **Q-4** in a concentration range from 6.25µM to 50µM. Fold change of fluorescence obtained in the presence of 5 mM NAC is given in brackets.

		**MES-SA R_5=H**	**MES-SA R_5=Cl**	**MES-SA/Dx5 R_5=H**	**MES-SA/Dx5 R_5=Cl**	**Cell free R_5=H**	**Cell free R_5=Cl**
**Q-1**	6.25 µM	0.965±0.12<br>(1.261±0.26)	1.147±0.32<br>(1.668±0.35)	1.006±0.15<br>(1.085±0.11)	1.203±0.32<br>(1.279±0.21)	1.098±0.50<br>(1.307±0.23)	1.175±0.32<br>(2.112±1.18)
	12.5 µM	0.799±0.12<br>(1.148±0.32)	1.119±0.30<br>(1.418±0.24)	0.720±0.21<br>(1.071±0.12)	1.291±0.48<br>(1.187±0.23)	0.891±0.24<br>(1.262±0.26)	1.214±0.43<br>(1.858±0.92)
	25 µM	0.722±0.22<br>(1.193±0.44)	1.071±0.35<br>(1.443±0.22)	0.713±0.21<br>(0.971±0.17)	1.355±0.46<br>(1.220±0.22)	0.917±0.35<br>(1.223±0.39)	1.271±0.88<br>(2.200±1.14)
	50 µM	0.748±0.21<br>(0.912±0.38)	0.891±0.34<br>(1.209±0.23)	0.725±0.18<br>(0.918±0.10)	1.206±0.49<br>(1.152±0.06)	1.094±0.65<br>(1.167±0.51)	1.011±0.50<br>(1.730±1.05)
**Q-2**	6.25 µM	1.598±0.32<br>(1.096±0.16)	1.896±0.32<br>(1.600±0.55)	1.863±0.81<br>(1.024±0.19)	2.594±0.87<br>(1.246±0.47)	1.266±0.45<br>(1.129±0.42)	1.920±1.42<br>(2.823±1.17)
	12.5 µM	1.792±0.53<br>(0.930±0.16)	2.226±0.39<br>(1.263±0.62)	2.324±1.25<br>(1.137±0.32)	3.214±0.86<br>(1.651±0.73)	1.297±0.61<br>(1.351±0.67)	2.196±1.53<br>(4.603±3.89)
	25 µM	2.101±0.86<br>(0.988±0.15)	2.086±0.66<br>(1.309±0.51)	3.348±2.35<br>(1.074±0.28)	13.870±1.70<br>(1.265±0.27)	1.308±0.69<br>(1.461±0.44)	2.732±2.28<br>(3.304±1.33)
	50 µM	2.454±1.39<br>(0.946±0.14)	2.609±0.77<br>(1.178±0.40)	4.103±2.67<br>(1.154±0.22)	4.697±3.24<br>(1.462±0.39)	2.350±2.19<br>(1.472±0.53)	2.435±1.51<br>(3.799±3.46)
**Q-3**	6.25 µM	1.288±0.24<br>(1.097±0.11)	1.672±0.27<br>(1.307±0.51)	1.449±0.46<br>(0.954±0.15)	1.975±0.50<br>(1.024±0.19)	0.823±0.17<br>(1.297±0.58)	2.088±1.94<br>(3.961±2.13)
	12.5 µM	1.372±0.22<br>(1.080±0.27)	1.861±0.40<br>(1.542±0.35)	1.400±0.18<br>(1.050±0.09)	1.954±0.42<br>(0.954±0.14)	1.114±0.53<br>(1.516±0.52)	1.760±0.97<br>(4.868±3.72)
	25 µM	1.464±0.39<br>(1.119±0.24)	1.812±0.27<br>(1.382±0.67)	1.586±0.22<br>(1.201±0.17)	2.714±0.84<br>(1.005±0.21)	1.103±0.45<br>(1.683±0.58)	2.156±1.85<br>(5.140±3.72)
	50 µM	1.753±0.37<br>(1.054±0.25)	2.075±0.68<br>(1.155±0.25)	1.786±0.36<br>(1.189±0.18)	3.077±1.29<br>(1.086±0.14)	1.076±0.38<br>(1.868±0.71)	1.849±1.53<br>(4.642±2.74)
**Q-4**	6.25 µM	1.431±0.39<br>(1.364±0.44)	1.207±0.20<br>(1.030±0.16)	1.282±0.35<br>(1.137±0.30)	1.238±0.28<br>(1.191±0.07)	1.684±2.02<br>(3.020±0.79)	0.924±0.45<br>(5.061±1.94)
	12.5 µM	1.118±0.14<br>(1.345±0.27)	1.190±0.14<br>(1.033±0.21)	1.177±0.22<br>(1.088±0.23)	1.396±0.35<br>(1.337±0.26)	1.437±0.88<br>(4.085±1.53)	0.864±0.34<br>(7.004±4.76)
	25 µM	1.260±0.20<br>(1.111±0.22)	1.187±0.29<br>(0.985±0.12)	1.344±0.27<br>(1.282±0.28)	1.340±0.26<br>(1.320±0.31)	1.648±1.19<br>(7.474±4.04)	0.745±0.29<br>(6.141±2.99)
	50 µM	1.328±0.29<br>(1.259±0.29)	1.151±0.19<br>(1.040±0.17)	1.373±0.35<br>(1.265±0.27)	1.286±0.30<br>(1.283±0.20)	2.248±1.31<br>(7.609±3.35)	1.462±1.42<br>(4.679±1.90)
